# Decoding SIGLEC12 in Bladder Cancer: In Silico Profiling of Expression, Tumor–Immune Interactions, and Prognostic Impact

**DOI:** 10.3390/medicina61111894

**Published:** 2025-10-22

**Authors:** Varsha Rathore, Wan-Wan Lin

**Affiliations:** 1 Chemical Biology and Molecular Biophysics, Taiwan International Graduate Program, Academia Sinica, Taipei 115201, Taiwan; s108080861@m108.nthu.edu.tw; 2 Institute of Biotechnology, College of Life Sciences and Medicine, National Tsing Hua University, Hsinchu 300044, Taiwan; 3 Department of Pharmacology, College of Medicine, National Taiwan University, Taipei 100233, Taiwan; 4 Graduate Institute of Medical Sciences, Taipei Medical University, Taipei 110, Taiwan

**Keywords:** bladder cancer, SIGLEC12, gene mutations, methylation, immunotherapy, drug resistance

## Abstract

*Background and Objectives:* Siglec-XII, encoded by SIGLEC12, is a unique sialic acid-binding immunoglobulin-like lectin. It lacks a highly conserved R122 residue for sialic acid recognition in humans. Although it is upregulated in bladder cancer (BCa), its role in tumorigenesis remains largely unexplored. This study aims to investigate the expression patterns of SIGLEC12 in BCa and its correlation with disease features. *Materials and Methods*: An integrated analysis of transcriptomic data and clinical profiles was conducted using various databases and tools, including UALCAN, GEPIA, TIMER, CAMOIP, and CPADs. The analyses encompassed SIGLEC12 expression, survival rates, immune infiltration levels, promoter methylation, and correlation with drug response. *Results*: SIGLEC12 expression was higher in both low-grade papillary and high-grade invasive non-papillary BCa. Higher SIGLEC12 expression resulting from low promoter hypomethylation was detected at the stage II-IV of BCa, and was unrelated to disease stages and metastatic stages. Elevated SIGLEC12 expression correlated with increased immune cell infiltration, higher expression of oncogenic and immune checkpoint blockade-related genes, and drug resistance signatures. Mutation analysis confirmed the absence of the canonical R122 missense mutation, indicating that the structural integrity and potential functionality of Siglec-XII are preserved in BCa. *Conclusions*: SIGLEC12 may have sialic acid recognition functions and serve as a potential early biomarker of BCa.

## 1. Introduction

Bladder cancer (BCa) is the most common malignant tumor of the urinary system, with the majority of cases arising from transitional cell carcinoma. Globally, BCa is among the ten most frequent cancers. According to GLOBOCAN 2020, it was responsible for over 210,000 deaths worldwide, occurring nearly three times more often in men than in women [1,2]. Clinically, BCa is categorized into non-muscle-invasive (NMIBC) and muscle-invasive (MIBC) types. NMIBC, which accounts for approximately three-quarters of new diagnoses, includes stages Ta, T1, and carcinoma in situ (Tis), confined to the mucosa or submucosa. In contrast, MIBC is defined by invasion into the muscularis propria (stage T2 and beyond) and has a poor prognosis due to its higher metastatic potential. Although most cases of NMIBC are less aggressive, high-grade forms remain at significant risk of progressing to MIBC [3]. Treatment strategies depend on stage and grade, ranging from transurethral resection and intravesical therapy for NMIBC to radical cystectomy, chemotherapy, radiotherapy, and immunotherapy for more advanced disease [4]. At the molecular level, MIBC has a greater mutational burden than NMIBC. Frequently altered genes in BCa include TERT, FGFR3, TP53, PIK3CA, and STAG2, as well as those regulating chromatin modification [5]. In Western countries, urothelial carcinoma constitutes over 90% of all bladder cancer cases, rendering it the most prevalent type. Urothelial carcinomas of the bladder originate as precursor lesions in the urothelium and progress through two stages: low-grade papillary carcinoma and high-grade invasive non-papillary lesions, such as carcinoma in situ. These clinical and molecular distinctions underscore the heterogeneity of BCa. Advances in single-cell analysis are expected to improve the molecular subtyping of both NMIBC and MIBC. Despite the availability of therapies, BCa imposes a considerable economic burden, underscoring the urgent need for reliable biomarkers to enable earlier diagnosis and more effective treatment [6,7].

Siglecs (sialic acid-binding immunoglobulin-like lectins) are protein receptors crucial in glyco-immunology and cancer progression. Tumors exploit hypersialylation to engage inhibitory Siglecs, which suppress immune responses and facilitate immune evasion, leading to poorer survival [8,9,10,11,12]. Research highlights both well-studied Siglecs (Siglec-1, -2, -3, -7, and -9) and less characterized members (Siglec-11 and Siglec-XII) as modulators of tumor–immune interactions [13]. Therapeutic strategies targeting Siglec–ligand pathways, including blocking antibodies, antibody–drug conjugates, bispecific antibodies, CAR-T, and glyco-engineered NK cells, have been developed to restore antitumor immunity [14,15,16]. Fifteen Siglec receptors exist in humans, with Siglec-XII having lost sialoglycan binding ability. Siglecs are categorized into conserved (Siglec-1, -2, -4, and -15) and CD33-related receptors (Siglec-3, 5–11, -XII, -14, and -16) [17]. Based on intracellular signaling domains, they are classified as inhibitory, activating, or non-signaling receptors. Inhibitory Siglec receptors contain a carbohydrate recognition domain that binds to sialoglycan ligands, C2-set domains, a transmembrane domain, and an intracellular domain with immunoreceptor tyrosine-based inhibitory motifs (ITIMs) and ITIM-like motifs that modulate signaling through Src-homology 2 domain (SH2)-containing phosphatase 1 (SHP1) and SHP2 phosphatases [18,19,20].

Among Siglecs, human Siglec-XII (encoded by the SIGLEC12 gene) represents an unusual and poorly characterized member. Unlike canonical Siglecs, Siglec-XII has lost a highly conserved arginine residue in its amino-terminal V-set domain during human evolution, rendering it incapable of binding sialic acids. This arginine to cysteine substitution, absent in “great ape” orthologs, is otherwise retained in chimpanzee Siglec-12, which recognizes Neu5Gc, a sialic acid variant lost in humans due to *CMAH* inactivation [21,22]. Moreover, the human protein contains two N-terminal V-set domains rather than the single domain typical of CD33-related Siglecs, further underscoring its atypical nature [23].

Despite the loss of canonical sialic acid binding, Siglec-XII still recruits Shp2-related oncogenic pathways and aids tumor growth and cancer progression [24,25]. Siglec-XII is detected in ~30–40% of normal epithelia, but ~80% of advanced carcinomas show strong expression of Siglec-XII [26]. Although the SIGLEC12 allele status did not predict prostate carcinoma incidence, forced expression of human Siglec-XII in a null prostate carcinoma cell line increases tumor growth in nude mice [24]. In addition, SIGLEC12 is highly expressed in renal cancer and is linked to primary tumor growth, increased immune cell infiltration in the tumor microenvironment, and poor survival in kidney renal clear cell carcinoma and kidney renal papillary cell carcinoma [27]. Similarly, SIGLEC12 expression is related to the pro-oncogenic and inflammation phenotypes in colorectal cancer [26]. Of note, over 80% of late-stage colorectal cancers have a functional SIGLEC12 allele, correlating with higher mortality [26,28].

In this study, we examined the expression of SIGLEC12 in BCa and normal tissue, identified oncogenic pathways that are upregulated in BCa expressing SIGLEC12, analyzed the impact on BCa patients’ response to therapy drugs, and addressed the predictive value of SIGLEC12 and its interaction with other genes in BCa cohorts.

## 2. Methods

### 2.1. Differential Gene Expression and Methylation Analysis

To investigate SIGLEC12 mRNA expression in BCa, the UALCAN database [29], which utilized TCGA transcriptomic data, was employed. The analysis involved comparing expression levels between normal and cancerous samples, including both papillary and non-papillary subtypes, and further explored various clinical subgroups such as TP53 mutation status, nodal metastasis stages (N0–N3), pathological stages I–IV, and patient gender. In UALCAN, mRNA expression levels were standardized as transcripts per million (TPM), and box-and-whisker plots illustrated the expression values across the sample groups, ranging from low to high. The platform calculated median values from these plots and used a Student’s *t*-test to assess up- or down-regulation by comparing normal and tumor samples. For statistical analysis, a Student’s *t*-test was conducted within UALCAN, with a significance threshold of *p* < 0.05. To adjust for multiple comparisons, the false discovery rate (FDR) method was applied.

Promoter methylation of SIGLEC12 was also assessed using UALCAN, which integrated TCGA DNA methylation data generated by the Illumina HumanMethylation450 BeadChip platform. β-Values (ranging from 0 = unmethylated to 1 = fully methylated) were calculated from CpG sites located within the promoter region (−1500 to +200 bp relative to the transcription start site) and displayed as box–whisker plots. Comparisons between normal and tumor samples, as well as clinical subgroups (TP53 mutation status, nodal metastasis stage N0–N3, pathological stages I–IV, and gender), were performed using Student’s *t*-test, with *p* < 0.05 considered significant.

### 2.2. Genetic Alteration Analysis

The cBioPortal database (https://www.cbioportal.org/, accessed on 3 June 2025) was used to assess alterations and mutation sites of SIGLEC12 in BCa using TCGA-BLCA PanCancer Atlas data. This platform provides a visualization of mutation distribution within the SIGLEC12 gene.

The Catalogue of Somatic Mutations in Cancer (COSMIC) database was employed to investigate SIGLEC12 mutations in BCa [30]. Through COSMIC, mutations were classified as missense, nonsense, synonymous, and frameshift, offering insight into their functional implications. The integration of cBioPortal and COSMIC data provided a thorough characterization of the SIGLEC12 mutation spectrum, highlighting sites of biological and clinical significance in BCa.

### 2.3. Functional Enrichment Analysis

To explore the relevant pathways and functional annotation associated with SIGLEC12 in bladder cancer (BCa), we conducted Gene Ontology (GO) and Kyoto Encyclopedia of Genes and Genomes (KEGG) enrichment analyses using the CAMOIP online tool [31]. These analyses enabled us to systematically identify biological processes, molecular functions, and cellular components in which SIGLEC12 and its related genes may be involved. KEGG pathway mapping further provided insights into the signaling cascades and oncogenic pathways that may be regulated by SIGLEC12. Further, we investigate the relationship between SIGLEC12 expression and other cancer-related genes in BCa, using the same CAMOIP tool. Patients in the TCGA-BLCA cohort were divided into two groups based on high and low SIGLEC12 expression, defined by median expression values, then assessed the differential expression of genes of interest, including HGF, PIK3R5, RHOC, RHOG, CEBPB, STAT2, FOXP3, IRF1, TGFB1, and FLT3LG, was assessed between the two groups. Boxplots were generated to visualize the distribution of expression levels, and statistical significance was determined using the default CAMOIP pipeline.

### 2.4. Survival Analysis

Survival analysis was conducted on the online database GEPIA [32]. BCa patients from the TCGA-BLCA cohort were divided into high and low groups based on the median expression level of SIGLEC12. Log-rank inspection and Mantel–Cox test were used to generate the survival curves, including overall survival and relapse-free survival. Hazard ratios (HR) with 95% confidence intervals were calculated to estimate the prognostic impact of SIGLEC12 expression.

### 2.5. Tumor Immune Infiltration and Immune Checkpoints Analysis

The analysis of immune cell infiltration in TCGA-BLCA samples was performed using two complementary deconvolution algorithms, CIBERSORT-ABS [33] and quanTIseq [34]. CIBERSORT-ABS utilizes *ν*-support vector regression (*ν*-SVR) to deconvolute bulk RNA-sequencing data, enabling the estimation of absolute fractions of distinct immune cell types. The analysis employed the LM22 signature matrix, implemented via the svm function in the R package e1071 [35]. Both the input expression data and the signature matrix were standardized to zero mean and unit variance, with LM22 markers selected for their high cell-type specificity and low collinearity. Standard normalization procedures were applied to reduce potential batch effects. In parallel, quanTIseq analysis was conducted using a constrained least squares regression approach with the TIL10 RNA-seq signature matrix, encompassing ten major immune cell types. Optional *ComBat/limma* harmonization was applied to correct for technical variability. Tumor purity was incorporated as a covariate, and partial Spearman correlation analysis was performed to assess the correlation between SIGLEC12 expression and immune cell infiltration levels.

Additionally, TIMER2.0 [36] was utilized to investigate correlations between SIGLEC12 and immune checkpoint genes (PDCD1, IDO1, CTLA4, LAG3, CD274). The TIMER algorithm applied a deconvolution approach to bulk RNA-seq data to estimate immune cell abundance and enabled the assessment of gene-gene co-expression through the Gene_Corr module. In this module, given a gene of interest and up to 20 additional genes, TIMER2.0 generated a heatmap of Spearman correlation coefficients across TCGA cancer types. The Benjamini–Hochberg (BH) procedure was used for multiple testing correction to control the false discovery rate (FDR), and correlations with FDR-adjusted *p* < 0.05 were considered statistically significant.

### 2.6. Drug Sensitivity Analysis

The relationship between SIGLEC12 expression and drug sensitivity in BCa was assessed using the CPADS web tool [37]. This platform utilized the pRRophetic ridge regression algorithm to predict the half-maximal inhibitory concentration (IC50) values of various therapeutic compounds based on gene expression data. In the tool, TCGA BLCA samples were automatically categorized into high and low SIGLEC12 expression groups, using the median expression level as a threshold. The platform then employed the Wilcoxon rank-sum test to compare the predicted IC50 values between these two expression groups, and the results were visualized as boxplots showing IC50 distributions, created internally with the ggplot2 package 3.2.1.

## 3. Results

### 3.1. SIGLEC12 Is Significantly Upregulated in BCa and Associates with Clinicopathological Features

**Figure 1:**
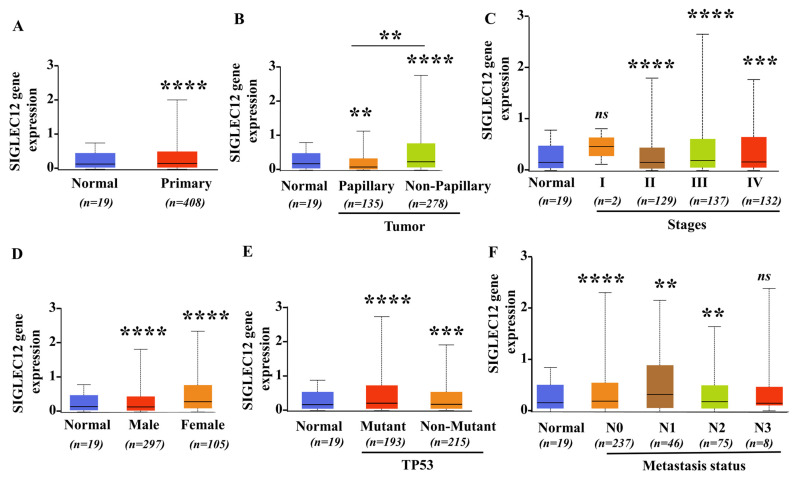
Expression of SIGLEC12 in BCa based on clinicopathological features. (**A**) Comparison of SIGLEC12 expression between normal bladder tissues (*n* = 19) and primary tumor tissues (*n* = 408). (**B**) Expression levels in papillary (*n* = 135) and non-papillary (*n* = 278) tumor subtypes compared with normal tissues (*n* = 21). (**C**) SIGLEC12 expression across tumor stages I (*n* = 2), II (*n* = 129), III (*n* = 137), and IV (*n* = 132) compared with normal tissues (*n* = 19). (**D**) Expression differences between male (*n* = 297) and female (*n* = 105) BCa patients compared with normal tissues (*n* = 19). (**E**) Expression comparison between TP53-mutant (*n* = 193) and non-mutant (*n* = 215) groups relative to normal tissues (*n* = 19). (**F**) SIGLEC12 expression across metastasis statuses: N0 (*n* = 237), N1 (*n* = 46), N2 (*n* = 75), and N3 (*n* = 8), compared with normal tissues (*n* = 19). Statistical analysis was performed using Student’s *t*-test. ** *p* < 0.01, *** *p* < 0.001, **** *p* < 0.0001. “ns”: non-significant.

Analysis of TCGA-BLCA transcriptomic data indicated that SIGLEC12 expression is significantly elevated in BCa compared to normal bladder tissue (*n* = 408 vs. n = 19, *p* < 0.0001) (Figure 1A). Stratification by histological subtypes indicates that both low-grade papillary (*n* = 135) and high-grade invasive non-papillary (*n* = 278) tumors exhibited higher expression levels relative to normal controls (*p* < 0.0001), with non-papillary tumors demonstrating significantly stronger expression than papillary tumors (*p* < 0.001) (Figure 1B). This suggests that up-regulation is not confined to a specific histological type. Across pathological stages, expression does not differ significantly at stage 1 (*n* = 2, underpowered), but becomes markedly elevated from stage 2 (*n* = 129, *p* < 0.0001) onwards, with the most pronounced increase observed at stage 3 (*n* = 137, *p* < 0.0001) and stage 4 (*n* = 132, *p* < 0.001) (Figure 1C), indicating a correlation with tumor progression and no significant difference among stages II–IV. Elevated SIGLEC12 expression in tumors is also observed irrespective of sex (male *n* = 297, female *n* = 105, *p* < 0.0001 vs. normal), with no significant differences between male and female (Figure 1D). Moreover, the elevated SIGLEC12 expression in tumors is irrespective of TP53 mutation status, as both mutant (*n* = 193) and wild type (*n* = 215) groups exhibited high expression level (*p* < 0.0001 vs. normal) (Figure 1E), highlighting that its dysregulation is a general characteristic of BCa. Analysis of nodal status indicated that SIGLEC12 expression is significantly increased in N0 (*n* = 237, *p* < 0.0001 vs. normal), N1 (*n* = 46, *p* < 0.001 vs. normal), and N2 (*n* = 75, *p* < 0.001 vs. normal) groups compared to normal tissue, while N3 (*n* = 8) showed no significant difference (Figure 1F). However, no significant difference was observed among the N0–N2 subgroups, likely due to the small sample size. Collectively, these findings demonstrate that SIGLEC12 up-regulation is an early and consistent event in BCa, which is associated with histological subtype, but independent of disease stages, metastasis, sex, or TP53 status.

### 3.2. Genetic Alterations of SIGLEC12 in BCa

**Figure 2:**
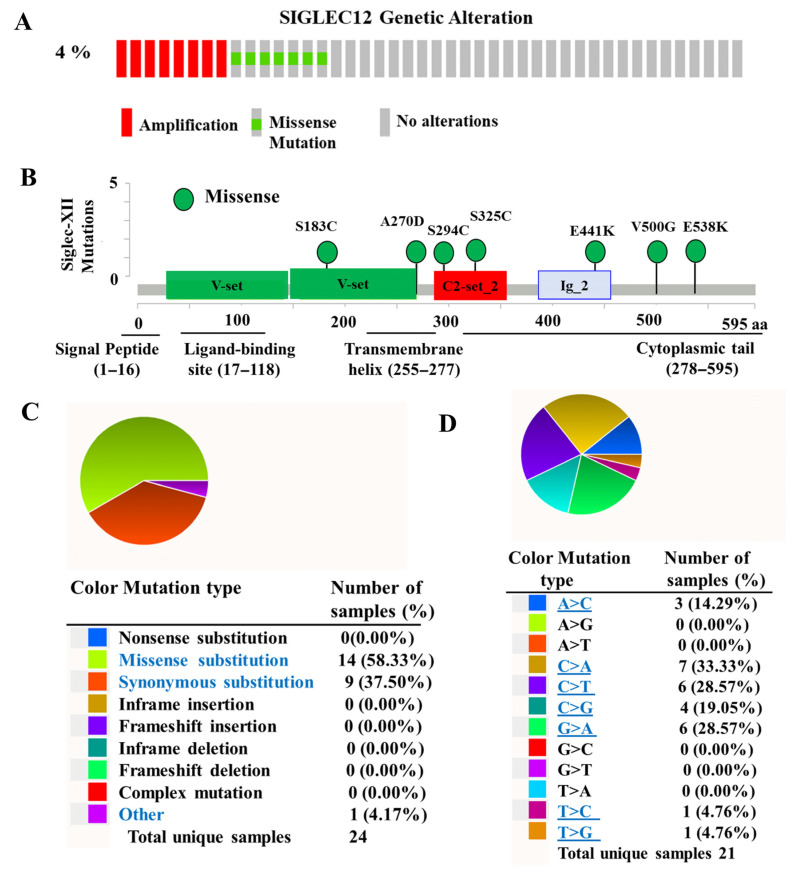
Genetic alterations and mutation profile of SIGLEC12 in BCa. (**A**) Overall frequency and type of genetic alterations in SIGLEC12, including amplification (red), missense mutation (green), and no alterations (grey), as identified in the TCGA-BLCA cohort using cBioPortal. (**B**) Lollipop plot showing the distribution of Siglec-XII mutations across protein domains, including V-set, C2-set, and Ig-like regions. Specific mutation sites are highlighted. (**C**) Pie chart depicting the proportion of mutation type, with missense substitutions. (**D**) Pie chart showing the base substitution spectrum of SIGLEC12 mutations.

SIGLEC12 is unusual among human Siglecs, because, unlike most family members, it is commonly inactivated in humans. In humans, SIGLEC12 underwent an R122C missense mutation in exon 1, eliminating the conserved arginine required for sialic acid binding [24]. In our analysis of TCGA datasets, however, we observed that approximately 4% of tumor samples retained somatic alterations in SIGLEC12, including missense mutation and copy number gains (Figure 2A). Mutation mapping identified missense variants at S183C, A270D, S294C, S325C, E441K, V500G, and E538K (Figure 2B), primarily distributed across the extracellular Ig-like domains except E441K. Importantly, the R122C missense mutation was not detected in this BCa tumor cohort. Instead, the mutations consisted predominantly of novel missense substitutions (58.3%), followed by synonymous changes (37.5%) (Figure 2C). At the nucleotide level, the most frequent substitutions were C > A (33.3%), C > T (28.6%), and G > A (28.6%) (Figure 2D). In total, 24 tumor samples carried non-synonymous mutations, while 21 samples carried base substitutions overall. These results suggest that tumor-associated mutations in SIGLEC12 differ from the canonical R122 pseudogenizing event, potentially preserving or altering functional activity in the tumor context.

### 3.3. Promoter Methylation Levels of SIGLEC12 Decreased in BCa

**Figure 3:**
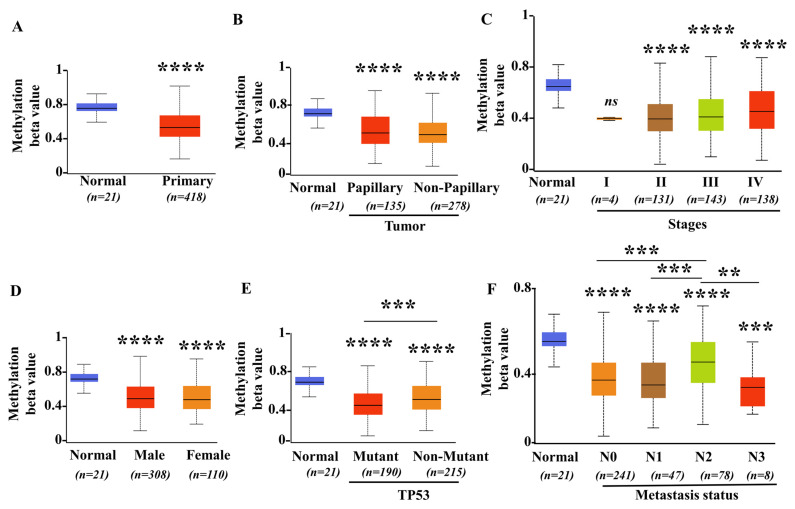
DNA methylation status of SIGLEC12 in BCa based on clinicopathological features. (**A**) Comparison of SIGLEC12 methylation β-values between normal bladder tissues (*n* = 21) and primary tumors (*n* = 418). (**B**) Methylation levels in papillary (*n* = 135) and non-papillary (*n* = 278) tumor subtypes compared with normal tissues (*n* = 21). (**C**) SIGLEC12 methylation across tumor stages I (*n* = 4), II (*n* = 131), III (*n* = 143), and IV (*n* = 138) compared with normal tissues (*n* = 21). (**D**) Methylation differences between male (*n* = 308) and female (*n* = 110) BCa patients relative to normal tissues (*n* = 21). (**E**) Comparison of methylation in TP53-mutant (*n* = 190) and non-mutant (*n* = 215) groups relative to normal tissues (*n* = 21). (**F**) Methylation profiles across nodal metastasis statuses: N0 (*n* = 241), N1 (*n* = 47), N2 (*n* = 78), and N3 (*n* = 8), compared with normal tissues (*n* = 21). Data are presented as boxplots of methylation β-values. Statistical analysis was performed using Student’s *t*-test. ** *p* < 0.01, *** *p* < 0.001, **** *p* < 0.0001. “ns”: non-significant.

To further investigate whether epigenetic mechanisms contribute to the aberrant upregulation of SIGLEC12 in BCa, we analyzed promoter methylation levels using TCGA data. Our results indicated consistent and robust hypomethylation of the SIGLEC12 promoter region in BCa samples (*n* = 418) compared to normal bladder tissues (*n* = 21) (Figure 3A), suggesting the loss of methylation may play a role in driving enhanced gene expression. Additionally, tumor histology analysis revealed that both papillary (*n* = 135) and non-papillary (*n* = 278) tumors exhibited lower methylation levels than normal tissue (*p* < 0.0001), with no major subtype-specific differences (Figure 3B), suggesting that hypomethylation is a general phenomenon in BCa. Across tumor stages, methylation levels were significantly decreased in stage II (*n* = 131), stage III (*n* = 143), and stage IV (*n* = 138) compared to normal tissues (*p* < 0.0001), with no significant difference among stages II-IV (Figure 3C). This indicates that promoter hypomethylation of SIGLEC12 is progressively reinforced during tumor progression. Stratification by sex showed both male (*n* = 308) and female (*n* = 110) patients exhibited significant promoter hypomethylation relative to normal tissues (*p* < 0.0001) with no significant differences between male and female (Figure 3D), confirming that this effect is not sex-specific. Comparison of TP53-mutant (*n* = 190) and non-mutant (*n* = 215) groups revealed consistent hypomethylation across both categories (*p* < 0.0001) relative to normal. We also observed a significant difference between wild-type and mutant groups (*p* < 0.001) with higher SIGLEC12 expression in non-mutant TP53 group than mutant group (Figure 3E). Analysis by metastatic status demonstrated progressive loss of promoter methylation from N0 (*n* = 241 vs. normal, *p* < 0.0001), N1 (*n* = 47 vs. normal, *p* < 0.0001), N2 (*n* = 78 vs. normal, *p* < 0.0001) to N3 (*n* = 8 vs. normal, *p* < 0.001). Significant differences were observed among subgroups N0 vs. N2 (*p* < 0.001), N1 vs. N2 (*p* < 0.001), and N2 vs. N3 (*p* < 0.01) (Figure 3F), reinforcing the notion that methylation loss correlates with more aggressive and metastatic disease. Taken together, these results strongly support that promoter hypomethylation is a key mechanism underlying the aberrant high expression of SIGLEC12 in BCa. The consistent observation across subtypes, sex, TP53 status, and advanced stages further suggests that methylation deregulation of SIGLEC12 may serve as a universal event in bladder tumorigenesis and progression.

### 3.4. GO and KEGG Pathway Enrichment Analysis of Genes Associated with SIGLEC12 Expression in BCa

**Figure 4:**
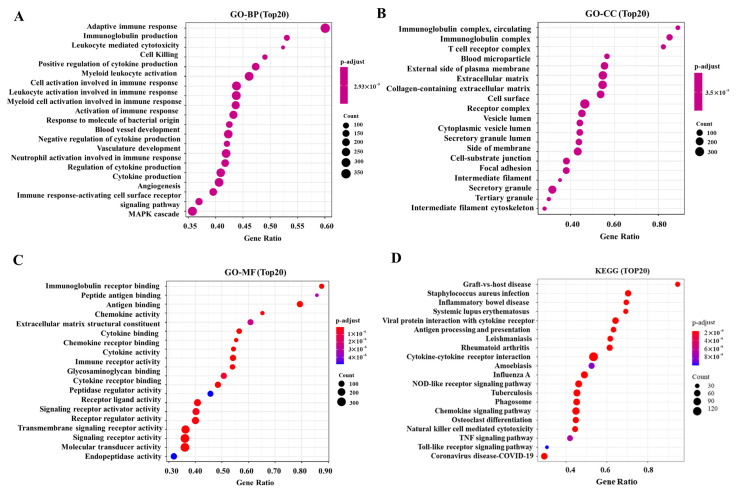
GO and KEGG enrichment analysis of SIGLEC12-associated genes in BCa. (**A**) Top 20 enriched Gene Ontology Biological Processes (GO-BP). (**B**) Top 20 enriched Gene Ontology Cellular Components (GO-CC). (**C**) Top 20 enriched Gene Ontology Molecular Functions (GO-MF). (**D**) Top 20 enriched Kyoto Encyclopedia of Genes and Genomes (KEGG) pathways. Bubble plots show the gene ratio on the *x*-axis, with bubble size representing the number of enriched genes and color intensity indicating the adjusted *p*-value.

When stratifying tumors by SIGLEC12 expression levels from the GO analysis, striking differences emerge in the immune- and inflammation-related signaling landscape. In the biological process (GO-BP) category, high SIGLEC12 expression was associated with strong enrichment of pathways such as adaptive immune response, immunoglobulin production, leukocyte-mediated cytotoxicity, and positive regulation of cytokine production (Figure 4A), suggesting that SIGLEC12 upregulation is coupled with enhanced immune cell activation and cytokine-driven signaling cascades. Consistently, in the cellular component (GO-CC) domain, the high-expression group showed enrichment in immuno-globulin complexes, T cell receptor complexes, vesicle lumens, and secretory granules (Figure 4B), pointing toward heightened immune receptor clustering and vesicle-mediated secretion. From the molecular function (GO-MF) perspective, SIGLEC12-high tumors were characterized by increased immunoglobulin receptor binding, antigen binding, chemokine activity, and cytokine receptor activity (Figure 4C), underscoring the role of SIGLEC12 in shaping receptor–ligand interactions and amplifying immune signaling. Finally, KEGG pathway enrichment further supported this trend, with high SIGLEC12 expression linked to diseases and processes involving hyperactive immune responses such as graft-versus-host disease, systemic lupus erythematosus, rheumatoid arthritis, inflammatory bowel disease, and cytokine–cytokine receptor interactions. Viral infection pathways (e.g., Influenza A, Coronavirus disease COVID-19) and innate immune signaling cascades (NOD-like receptor, Toll-like receptor, and TNF signaling) were also prominently enriched (Figure 4D). Collectively, these results suggest that elevated SIGLEC12 expression promotes an immune-activated and inflammatory microenvironment, whereas the low-expression state may correspond to reduced immune engagement and impaired antigen presentation, possibly influencing tumor immune evasion and therapy responsiveness.

### 3.5. Correlation Between SIGLEC12 Expression and Immune Cell Composition in BCa

**Figure 5:**
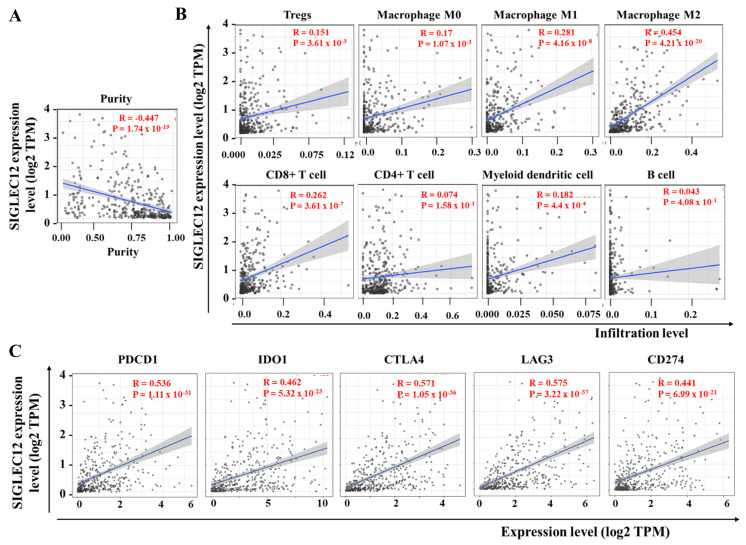
Correlation of SIGLEC12 expression with immune infiltration and immune checkpoint molecules in BCa. (**A**) Association between SIGLEC12 expression and tumor purity. (**B**) Correlation between SIGLEC12 expression and infiltration levels of various immune cells, including Tregs, macrophages (M0, M1, M2), CD8^+^ T cells, CD4^+^ T cells, myeloid dendritic cells, and B cells. (**C**) Correlation between SIGLEC12 expression and major immune checkpoint molecules, including PDCD1, IDO1, CTLA4, LAG3, and CD274. Scatter plots display Spearman correlation coefficients (Rho, R) and corresponding *p*-values. Shaded regions represent 95% confidence intervals for the fitted regression line.

Immune cell infiltration plays a crucial role in the development, progression, and prognosis of tumors. Here, we observed the correlation between SIGLEC12 expression and tumor purity (Rho = −0.447, *p* = 1.74 × 10^−19^) (Figure 5A), indicating higher expression in more immune-infiltrated tumors. As shown in Figure 5B, SIGLEC12 levels were correlated positively with pro-tumor immune subsets, Tregs (Rho = 0.151, *p* = 3.61 × 10^−3^), M0 macrophages (Rho = 0.170, *p* = 1.07 × 10^−3^), and M2 macrophages (Rho = 0.454, *p* = 4.20 × 10^−20^). Notably, M2 macrophages demonstrated the strongest association. SIGLEC12 was also associated with M1 macrophages (Rho = 0.281, *p* = 4.16 × 10^−8^), CD8^+^ T cells (Rho = 0.262, *p* = 3.61 × 10^−7^), and myeloid dendritic cells (Rho = 0.182, *p* = 4.40 × 10^−4^). CD4^+^ T cells showed no statistically significant correlation (Rho = 0.074, *p* = 0.158), and B cells displayed a weak, non-significant trend (Rho = 0.043, *p* = 0.408) (Figure 5B). After assessing the correlation of SIGLEC12 with eight immune cell types using CIBERSORT, we validated the findings with the quanTIseq platform to ensure robustness (Table 1). Both methods employ distinct computational algorithms, thereby strengthening the reliability of the observed associations. These results suggest that SIGLEC12 expression is linked to a complex immune contexture, with enrichment in both pro-tumor and anti-tumor immune populations. However, the strongest associations occur with immunosuppressive M2 macrophages.

Furthermore, we analyzed the SIGLEC12 association with various inhibitory immune checkpoint genes in BCa. In this aspect, SIGLEC12 expression was demonstrated to have significant positive correlations with checkpoint molecules, such as PDCD1 (Rho = 0.536, *p* = 1.11 × 10^−31^), IDO1 (Rho = 0.462, *p* = 5.32 × 10^−23^), CTLA4 (Rho = 0.571, *p* = 1.05 × 10^−36^), and LAG3 (Rho = 0.575, *p* = 3.22 × 10^−37^), along with CD274, commonly known as PD-L1 (Rho = 0.441, *p* = 6.99 × 10^−21^) (Figure 5C). Notably, the strongest correlations were observed with CTLA4 and LAG3, both of which play crucial roles in T cell exhaustion and are primary targets of ICB therapies. These results suggest that SIGLEC12 expression is intricately linked to a diverse range of immune inhibitory pathways.

### 3.6. SIGLEC12 Expression Correlates with Oncogenic Signaling but Not Survival Outcome in BCa

**Figure 6:**
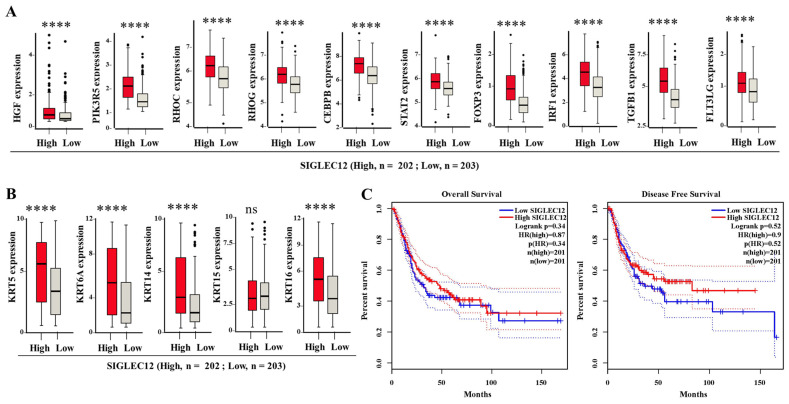
Association of SIGLEC12 expression with oncogenic gene signatures and survival outcomes in BCa. (**A**) Differential expression analysis of oncogenic and immunoregulatory genes in patients with high vs. low SIGLEC12 expression. Genes including HGF, PIK3R5, PIROC, CEBPB, STAT2, FOXP3, IRF1, TGFB1, and FLT3LG were shown. (**B**) Correlation of SIGLEC12 expression with keratin family members (KRT5, KRT6A, KRT14, KRT15, KRT16) in BCa. (**C**) Kaplan–Meier survival curves comparing high and low SIGLEC12 expression groups. Statistical analysis was performed using Student’s *t*-test. **** *p* < 0.0001. “ns”: non-significant.

We next evaluated the transcriptional landscape associated with differential SIGLEC12 expression. Patients were stratified into high (*n* = 202) and low (*n* = 203) SIGLEC12 expression groups. As shown in Figure 6A, high SIGLEC12 expression was significantly associated with increased expression of several oncogenic and immunoregulatory genes, including HGF, PIK3R5, RHOC, RHOG, CEBPB, STAT2, FOXP3, IRF1, TGFB1, and FLT3LG. These findings indicate that elevated SIGLEC12 expression is linked to tumor-promoting pathways, particularly those involving growth factor signaling, PI3K pathway activation, and immunosuppressive transcription factors. Moreover, we assessed the relationship between SIGLEC12 expression and keratin family members in BCa; some of which were shown to correlate with tumor progression [38]. SIGLEC12 expression showed strong associations with KRT5, KRT6A, KRT14, and KRT16, but not KRT15.

We also assessed the prognostic relevance of SIGLEC12 expression using Kaplan–Meier survival analysis. Patients with high SIGLEC12 expression showed no significant difference in overall survival compared to those with low expression (*p* = 0.34, HR = 0.87) (Figure 6C, left panel). Likewise, disease-free survival did not differ significantly between the two groups (*p* = 0.52, HR = 0.9) (Figure 6C, right panel). Thus, while SIGLEC12 overexpression is associated with pro-tumorigenic gene expression signatures, it cannot independently predict patient survival outcomes in this cohort.

### 3.7. High SIGLEC12 Expression Is Associated with Drug Resistance

**Figure 7:**
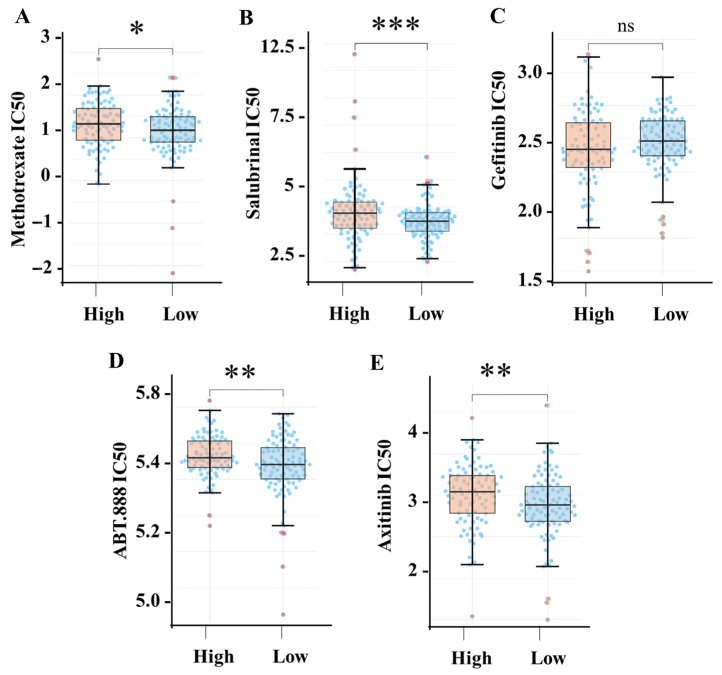
Association between SIGLEC12 expression and drug response. Boxplots show IC50 values for (**A**) methotrexate, (**B**) salubrinal, (**C**) gefitinib, (**D**) ABT-888 (veliparib), and (**E**) axitinib in TCGA-BLCA between high and low SIGLEC12 expression. * *p* < 0.05, ** *p* < 0.01, *** *p* < 0.001, “ns”: non-significant.

Analysis of drug response profiles revealed a consistent association between elevated SIGLEC12 expression and increased drug resistance. Specifically, TCGA patients with high SIGLEC12 expression exhibited significantly higher IC50 values for methotrexate (Figure 7A, *p* < 0.05), salubrinal (Figure 7B, *p* < 0.001), ABT-888 (veliparib) (Figure 7D, *p* < 0.01), and axitinib (Figure 7E, *p* < 0.01) when compared with the low SIGLEC12 group. These findings indicate that high SIGLEC12 expression diminishes sensitivity to both classical chemotherapeutics and targeted agents, including an antimetabolite (methotrexate), a PARP inhibitor (ABT-888), an angiogenesis inhibitor (axitinib), and an ER stress modulator (salubrinal). In contrast, no significant difference in IC50 values was observed for gefitinib (Figure 7C), suggesting that SIGLEC12 does not affect EGFR inhibitor response. Together, these results demonstrate that SIGLEC12 overexpression is broadly correlated with reduced therapeutic susceptibility across multiple drug classes, highlighting its potential role in drug resistance.

## 4. Discussion

The Siglec family has emerged as immune checkpoint-like regulators in cancer, with Siglec-7 and Siglec-9 associated with prostate cancer [39]. Siglec-15 is identified as a therapeutic target in tumor-associated macrophages and various types of cancer cells, particularly in colon cancer [40,41], and Siglec-10 was recently found to inhibit macrophage phagocytosis through integrin interactions [42]. SIGLEC12 has been regarded as a non-functional pseudogene in humans due to a missense mutation (R122C) in exon 1 of the V-set domain, disrupting the arginine residue required for sialic acid binding [24]. The role of SIGLEC12 in BCa remains unexplored, despite potential involvement in tumorigenesis in prostate, colorectal, and renal cancers [24,26,27,28]. In this study, we demonstrate that SIGLEC12 expression is highly increased at the early stage of BCa due to promoter hypomethylation and is associated with the immune context and oncogenic pathways in the tumor microenvironment, as well as drug resistance. We show for the first time the absence of the R122C missense mutation in Siglec-XII, suggesting the possible function of Siglec-XII in BCa.

Although several risk factors in BCa have been documented [43], and high SIGLEC12 expression levels are shown in colorectal cancer [26] and renal cancer [27], the role of SIGLEC12 in BCa has not been documented. Our study indicates that SIGLEC12 is significantly expressed in BCa tissues, with lower expression in normal bladder tissues (Figure 1A), indicating that SIGLEC12 may serve as a potential biomarker. Higher SIGLEC12 expression levels occurred in both low-grade papillary and high-grade invasive non-papillary subtypes (Figure 1B), and advanced stages (II–IV) (Figure 1C). Nevertheless, SIGLEC12 expression is unrelated to metastatic stages (Figure 1F), overall survival (Figure 6C), and disease-free survival (Figure 6C) in BCa patients. These findings suggest that SIGLEC12 may be an early potential biomarker of BCa, while its other clinical application needs further investigation.

In this study, we observed that the high SIGLEC12 expression in BCa is associated with gene amplification and mutation. In BCa tumors, SIGLEC12 is expressed with 7 mutation sites (Figure 2B) without the R122C missense inactivating mutation (Figure 2B), indicating the structural integrity and possible functional capacity of Siglec-XII in BCa. This is in agreement with a previous study in colorectal cancer, where SIGLEC12 has a functional allele [26]. Moreover, the newly identified mutation sites at S183C, A270D, S294C, S325C, E441K, V500G, and E538K are present in different structural domains of Siglec-XII with important receptor functions [44,45]. In this aspect, the V-set domain containing S183C and A270D is responsible for the recognition of sialic acid residues on glycoproteins. The C2-set_2 domain containing S294C and S325C is a structure Ig-like domain that helps maintain the extracellular conformation of the receptor. The Ig_2 domain containing E441K is the second Ig-like domain in SIGLEC12 and is involved in structural stabilization and Protein–Protein Interactions. The cytoplasmic tail containing V500G and E538K constitutes the ITIM sequence and plays a crucial role in cellular signaling and the functions of Siglec-XII receptor. The biological functions of these mutations in SIGLEC12 are worthy of investigation in the future.

DNA hypomethylation is recognized as an oncogene activation mechanism, particularly in tumor progression. Until now, the epigenetic regulation of SIGLE12 has been less investigated. Only a few reports showed the unmethylation in the SIGLEC12 promoters in Kaposi’s sarcoma [46] and oral cancer [47]. In this study, we identified epigenetic hypomethylation as a regulatory mechanism for SIGLEC12 overexpression, and methylation deregulation of SIGLEC12 may serve as a universal event in bladder tumorigenesis and progression. Our findings align with research on SIGLEC15, where hypomethylation links to abnormal expression in lung, colon, and kidney cancer [48]. The correlation between reduced methylation and higher SIGLEC12 expression supports a model where epigenetic deregulation enables re-expression of an otherwise silent Siglec in malignant urothelium, positioning SIGLEC12 can be a potential target for epigenetic therapies. In this study, the correlation of SLGLEC12 with TP53 mutation status is noteworthy. TP53 mutations and inactivation are key to bladder carcinogenesis, genomic instability, and poor prognosis [49]. However, there are currently no direct reports linking p53 regulation to DNA methylation of SIGLEC genes. Although TP53 status (wild type or mutant) does not alter the SIGLEC12 expression (Figure 1E), it affects the promoter methylation of SIGLEC12 (Figure 3E). It is suggested that other epigenetic or non-epigenetic mechanisms beyond TP53 are involved in the regulation of SIGLEC12 expression.

Immune/inflammation pathways are crucial for cancer progression. Our data suggest that elevated SIGLEC12 expression promotes an immune-activated and inflammatory microenvironment, whereas the low-expression state may correspond to reduced immune engagement and impaired antigen presentation, providing new insights into SIGLEC12 and immune dysregulation. High SIGLEC12 expression correlates with biological processes central to adaptive immunity (Figure 4A), leukocyte activation (Figure 4A), cytokine regulation (Figure 4C), and NK-cell cytotoxicity (Figure 4D). KEGG analysis reveals associations with cytokine–cytokine receptor interactions, Toll-like receptor signaling, NOD-like receptor signaling, and viral infection pathways (Figure 4D). These findings suggest SIGLEC12 expression reflects tumor biology and may shape the tumor–immune interface. In this aspect, our results suggest that SIGLEC12 expression is linked to a complex immune contexture, with enrichment in both pro-tumor and anti-tumor immune populations (Figure 5B). However, the strongest associations occur with immunosuppressive M2 macrophages.

In addition, SIGLEC12 expression correlates with FOXP3, IRF1, and TGFB1, key immune regulators. FOXP3 is the lineage-defining factor for Tregs, which suppress antitumor immunity [50]; IRF1 is a transcription factor in interferon signaling that promotes immune escape [51]; and TGFB1 is an immunosuppressive cytokine with roles in epithelial–mesenchymal transition and metastasis [52,53]. These associations suggest that SIGLEC12 may contribute to an immunosuppressive tumor microenvironment. While SIGLEC12’s sialic acid-binding capacity is compromised in many individuals, its expression in BCa may impact immune recognition through alternative binding mechanisms. That SIGLEC12 contributes to immune evasion independent of canonical ligand binding may distinguish it from other Siglecs and warrant investigation. Our findings align with reports that SIGLEC12 expression in other cancers, as we mentioned above, predicts worse outcomes. We identified associations between SIGLEC12 expression and core oncogenic pathways. Co-expression analysis highlights genes like PIK3R5 (PI3K–AKT signaling), CEBPB (linking inflammation to tumor progression), and STAT2 (JAK–STAT amplification). These associations position SIGLEC12 within networks of signaling cascades that sustain tumor growth. Taken together, all the correlation data need validation in functional assays and detailed mechanistic exploration in the future.

In recent years, immunotherapies, especially immune checkpoint blockade therapy, have arisen as an auspicious approach to treat various cancers, including BCa [54,55]. Interestingly, our analysis suggests that SIGLEC12 expression is intricately linked to a diverse range of immune inhibitory pathways, and elevated SIGLEC12 levels may contribute to an immunosuppressive tumor microenvironment in BCa. SIGLEC12 expression demonstrates a significant correlation with immune checkpoint molecules, including PDCD1, IDO1, CTLA4, LAG3, and CD274. Furthermore, correlations with HGF suggest that SIGLEC12 is involved in angiogenesis and tumor progression, as HGF promotes motility and invasion [56]. Furthermore, our findings indicate that SIGLEC12 expression parallels some keratin markers, suggesting that it may serve as an additional indicator of tumor phenotype and prognosis in BCa. The expression association of SIGLEC12 was found for KRT5, 6A, 14, and 16. KRT5 is a characteristic feature of the chemosensitive basal subtype of MIBC, and it is linked to the aggressive basal subtype [57]. KRT6A and KRT14 are linked to aggressive disease and poor survival in BCa [58,59]. Although KRT15 is routinely applied to classify basal versus luminal subtypes [60], its expression is not in parallel to SIGLEC12.

However, SIGLEC12 expression did not significantly alter overall survival or disease-free survival in the BCa cohort; one reason is due to limited sample sizes, treatment variability, and tumor subtype differences. Gene expression analysis results indicate that SIGLEC12 may primarily function as a co-driver of disease progression rather than an independent prognostic factor. The absence of survival impact may be due to limited sample size and clinical heterogeneity, including treatment variability and tumor subtype differences. Future validation in larger cohorts and functional assays will determine its full translational value. Despite this, the information provided by this study suggests that SIGLEC12 may hold potential as a biomarker for BCa, potentially guiding stratification of aggressive BCa subtypes and therapeutic resistance profiles.

It is noteworthy to mention the significant association of SIGLEC12 with drug resistance. The increased expression of SIGLEC12 appears to correlate with elevated IC50 values for several agents, including methotrexate (an antimetabolite) [61], PARP inhibitors (ABT-888) [62], axitinib (a VEGFR tyrosine kinase inhibitor) [63], and salubrinal (an ER stress modulator) [64]. These observations suggest that SIGLEC12 expression may confer broad resistance across various drug classes. Interestingly, the efficacy of gefitinib seems to remain unaffected, which may indicate that EGFR-driven pathways operate independently of SIGLEC12 activity. Future experimental studies, such as functional assays with SIGLEC12 overexpression or knockdown, are needed to validate causal relationships and clarify the underlying mechanisms for drug resistance.

## 5. Conclusions

This study offers insight into an underappreciated Siglec as a pivotal molecular component in disease biology. We highlight SIGLEC12 as a potential marker in BCa, whose high expression resulting from low hypomethylation is associated with immune contexture and drug resistance signatures. Through epigenetic deregulation and absence of canonical mutation, SIGLEC12 is transcriptionally upregulated, associated with oncogenic and angiogenic pathways, and influences the immune microenvironment towards immunosuppression. Its expression is associated with aggressive features and therapeutic resistance, emphasizing its significance in prognosis and treatment. These findings suggest SIGLEC12 is not merely an evolutionary relic but can be a clinically relevant immuno-oncology target. Future research is required for mechanistic validation, functional assays, clinical stratification, and translational development to understand SIGLEC12 in Siglec-targeted therapies in BCa.

## Figures and Tables

**Table 1:** Correlation between SIGLEC12 expression and immune cells using quanTIseq.

Cell Type	Rho (R)	*p*-Value
Tregs	0.293	<0.001
Macrophage M1	0.361	<0.001
Macrophage M2	0.256	<0.001
CD8+ T cell	0.225	<0.001
CD4+ T cell	−0.04	0.4
Myeloid dendritic cell	−0.175	<0.001
B cell	0.03	0.05

## Data Availability

All datasets analyzed in this study were obtained from publicly available databases and open-access tools. The data can be accessed through the respective websites and platforms cited in the manuscript. No new datasets were generated during this study.

## References

[1] Bray F., Laversanne M., Sung H., Ferlay J., Siegel R.L., Soerjomataram I., Jemal A. (2024). Global Cancer Statistics 2022: GLOBOCAN Estimates of Incidence and Mortality Worldwide for 36 Cancers in 185 Countries. CA A Cancer J. Clin..

[2] Toren P., Wilkins A., Patel K., Burley A., Gris T., Kockelbergh R., Lodhi T., Choudhury A., Bryan R.T. (2024). The Sex Gap in Bladder Cancer Survival—A Missing Link in Bladder Cancer Care?. Nat. Rev. Urol..

[3] Cassell A., Yunusa B., Jalloh M., Mbodji M.M., Diallo A., Ndoye M., Diallo Y., Labou I., Niang L., Gueye S.M. (2019). Non-Muscle Invasive Bladder Cancer: A Review of the Current Trend in Africa. World J. Oncol..

[4] Abd El-Salam M.A., Smith C.E.P., Pan C. (2022). Insights on Recent Innovations in Bladder Cancer Immunotherapy. Cancer Cytopathol..

[5] Dyrskjøt L., Hansel D.E., Efstathiou J.A., Knowles M.A., Galsky M.D., Teoh J., Theodorescu D. (2023). Bladder Cancer. Nat. Rev. Dis. Primers.

[6] Czerniak B., Dinney C., McConkey D. (2016). Origins of Bladder Cancer. Annu. Rev. Pathol. Mech. Dis..

[7] Guo C.C., Czerniak B. (2019). Bladder Cancer in the Genomic Era. Arch. Pathol. Lab. Med..

[8] Beatson R., Tajadura-Ortega V., Achkova D., Picco G., Tsourouktsoglou T.-D., Klausing S., Hillier M., Maher J., Noll T., Crocker P.R. (2016). The Mucin MUC1 Modulates the Tumor Immunological Microenvironment through Engagement of the Lectin Siglec-9. Nat. Immunol..

[9] Büll C., Boltje T.J., Van Dinther E.A.W., Peters T., De Graaf A.M.A., Leusen J.H.W., Kreutz M., Figdor C.G., Den Brok M.H., Adema G.J. (2015). Targeted Delivery of a Sialic Acid-Blocking Glycomimetic to Cancer Cells Inhibits Metastatic Spread. ACS Nano.

[10] Chiang C., Wang C., Chang H., More S.V., Li W., Hung W. (2010). A Novel Sialyltransferase Inhibitor AL10 Suppresses Invasion and Metastasis of Lung Cancer Cells by Inhibiting Integrin-mediated Signaling. J. Cell. Physiol..

[11] Jandus C., Simon H.-U., Von Gunten S. (2011). Targeting Siglecs—A Novel Pharmacological Strategy for Immuno- and Glycotherapy. Biochem. Pharmacol..

[12] Läubli H., Pearce O.M.T., Schwarz F., Siddiqui S.S., Deng L., Stanczak M.A., Deng L., Verhagen A., Secrest P., Lusk C. (2014). Engagement of Myelomonocytic Siglecs by Tumor-Associated Ligands Modulates the Innate Immune Response to Cancer. Proc. Natl. Acad. Sci. USA.

[13] Lim J., Sari-Ak D., Bagga T. (2021). Siglecs as Therapeutic Targets in Cancer. Biology.

[14] Daly J., Sarkar S., Natoni A., Stark J.C., Riley N.M., Bertozzi C.R., Carlsten M., O’Dwyer M.E. (2022). Targeting Hypersialylation in Multiple Myeloma Represents a Novel Approach to Enhance NK Cell–Mediated Tumor Responses. Blood Adv..

[15] Sterner R.C., Sterner R.M. (2021). CAR-T Cell Therapy: Current Limitations and Potential Strategies. Blood Cancer J..

[16] Tian Z., Liu M., Zhang Y., Wang X. (2021). Bispecific T Cell Engagers: An Emerging Therapy for Management of Hematologic Malignancies. J. Hematol. Oncol..

[17] Bornhöfft K.F., Goldammer T., Rebl A., Galuska S.P. (2018). Siglecs: A Journey through the Evolution of Sialic Acid-Binding Immunoglobulin-Type Lectins. Dev. Comp. Immunol..

[18] Avril T., Freeman S.D., Attrill H., Clarke R.G., Crocker P.R. (2005). Siglec-5 (CD170) Can Mediate Inhibitory Signaling in the Absence of Immunoreceptor Tyrosine-Based Inhibitory Motif Phosphorylation. J. Biol. Chem..

[19] Crocker P.R., Paulson J.C., Varki A. (2007). Siglecs and Their Roles in the Immune System. Nat. Rev. Immunol..

[20] Bärenwaldt A., Läubli H. (2019). The Sialoglycan-Siglec Glyco-Immune Checkpoint—A Target for Improving Innate and Adaptive Anti-Cancer Immunity. Expert. Opin. Ther. Targets.

[21] Khan N., De Manuel M., Peyregne S., Do R., Prufer K., Marques-Bonet T., Varki N., Gagneux P., Varki A. (2020). Multiple Genomic Events Altering Hominin SIGLEC Biology and Innate Immunity Predated the Common Ancestor of Humans and Archaic Hominins. Genome Biol. Evol..

[22] Angata T., Varki N.M., Varki A. (2001). A Second Uniquely Human Mutation Affecting Sialic Acid Biology. J. Biol. Chem..

[23] McDonough C.W., Gong Y., Padmanabhan S., Burkley B., Langaee T.Y., Melander O., Pepine C.J., Dominiczak A.F., Cooper-DeHoff R.M., Johnson J.A. (2013). Pharmacogenomic Association of Nonsynonymous SNPs in *SIGLEC12*, *A1BG*, and the Selectin Region and Cardiovascular Outcomes. Hypertension.

[24] Mitra N., Banda K., Altheide T.K., Schaffer L., Johnson-Pais T.L., Beuten J., Leach R.J., Angata T., Varki N., Varki A. (2011). SIGLEC12, a Human-Specific Segregating (Pseudo) Gene, Encodes a Signaling Molecule Expressed in Prostate Carcinomas. J. Biol. Chem..

[25] Yu Z., Lai C.-M., Maoui M., Banville D., Shen S.-H. (2001). Identification and Characterization of S2V, a Novel Putative Siglec That Contains Two V Set Ig-like Domains and Recruits Protein-Tyrosine Phosphatases SHPs. J. Biol. Chem..

[26] Siddiqui S.S., Vaill M., Do R., Khan N., Verhagen A.L., Zhang W., Lenz H.-J., Johnson-Pais T.L., Leach R.J., Fraser G. (2021). Human-Specific Polymorphic Pseudogenization of SIGLEC12 Protects against Advanced Cancer Progression. FASEB Bioadv.

[27] Ogbodo A.K., Mustafov D., Arora M., Lambrou G.I., Braoudaki M., Siddiqui S.S. (2024). Analysis of SIGLEC12 Expression, Immunomodulation and Prognostic Value in Renal Cancer Using Multiomic Databases. Heliyon.

[28] Cuello H.A., Sinha S., Verhagen A.L., Varki N., Varki A., Ghosh P. (2024). Human-Specific Elimination of Epithelial Siglec-XII Suppresses the Risk of Inflammation Driven Colorectal Cancers. JCI Insight.

[29] Chandrashekar D.S., Bashel B., Balasubramanya S.A.H., Creighton C.J., Ponce-Rodriguez I., Chakravarthi B.V.S.K., Varambally S. (2017). UALCAN: A Portal for Facilitating Tumor Subgroup Gene Expression and Survival Analyses. Neoplasia.

[30] Forbes S.A., Beare D., Gunasekaran P., Leung K., Bindal N., Boutselakis H., Ding M., Bamford S., Cole C., Ward S. (2015). COSMIC: Exploring the World’s Knowledge of Somatic Mutations in Human Cancer. Nucleic Acids Res..

[31] Lin A., Qi C., Wei T., Li M., Cheng Q., Liu Z., Luo P., Zhang J. (2022). CAMOIP: A Web Server for Comprehensive Analysis on Multi-Omics of Immunotherapy in Pan-Cancer. Brief. Bioinform..

[32] Tang Z., Li C., Kang B., Gao G., Li C., Zhang Z. (2017). GEPIA: A Web Server for Cancer and Normal Gene Expression Profiling and Interactive Analyses. Nucleic Acids Res..

[33] Sturm G., Finotello F., Petitprez F., Zhang J.D., Baumbach J., Fridman W.H., List M., Aneichyk T. (2019). Comprehensive Evaluation of Transcriptome-Based Cell-Type Quantification Methods for Immuno-Oncology. Bioinformatics.

[34] Plattner C., Finotello F., Rieder D. (2020). Deconvoluting Tumor-Infiltrating Immune Cells from RNA-Seq Data Using quanTIseq. Methods Enzymol..

[35] White B.S., de Reyniès A., Newman A.M., Waterfall J.J., Lamb A., Petitprez F., Lin Y., Yu R., Guerrero-Gimenez M.E., Domanskyi S. (2024). Community Assessment of Methods to Deconvolve Cellular Composition from Bulk Gene Expression. Nat. Commun..

[36] Li T., Fan J., Wang B., Traugh N., Chen Q., Liu J.S., Li B., Liu X.S. (2017). TIMER: A Web Server for Comprehensive Analysis of Tumor-Infiltrating Immune Cells. Cancer Res..

[37] Li K., Yang H., Lin A., Xie J., Wang H., Zhou J., Carr S.R., Liu Z., Li X., Zhang J. (2024). CPADS: A Web Tool for Comprehensive Pancancer Analysis of Drug Sensitivity. Brief. Bioinform..

[38] Crocetto F., Amicuzi U., Musone M., Magliocchetti M., Di Lieto D., Tammaro S., Pastore A.L., Fuschi A., Falabella R., Ferro M. (2025). Liquid Biopsy: Current Advancements in Clinical Practice for Bladder Cancer. J. Liq. Biopsy.

[39] Wen R.M., Stark J.C., Marti G.E.W., Fan Z., Lyu A., Garcia Marques F.J., Zhang X., Riley N.M., Totten S.M., Bermudez A. (2024). Sialylated Glycoproteins Suppress Immune Cell Killing by Binding to Siglec-7 and Siglec-9 in Prostate Cancer. J. Clin. Investig..

[40] Ma Z., Hao X., Qu S., Zhang Q., Luo J., Li H., Liu J., Dai W., Li J., Gu S. (2025). Siglec-15 Antibody–GM-CSF Chimera Suppresses Tumor Progression via Reprogramming Tumor-Associated Macrophages. J. Immunother. Cancer.

[41] Wang J., Sun J., Liu L.N., Flies D.B., Nie X., Toki M., Zhang J., Song C., Zarr M., Zhou X. (2019). Siglec-15 as an Immune Suppressor and Potential Target for Normalization Cancer Immunotherapy. Nat. Med..

[42] Wang C., He L., Peng J., Lu C., Zhang M., Qi X., Zhang M., Wang Y. (2024). Identification of Siglec-10 as a New Dendritic Cell Checkpoint for Cervical Cancer Immunotherapy. J. Immunother. Cancer.

[43] Barone B., Finati M., Cinelli F., Fanelli A., Del Giudice F., De Berardinis E., Sciarra A., Russo G., Mancini V., D’Altilia N. (2023). Bladder Cancer and Risk Factors: Data from a Multi-Institutional Long-Term Analysis on Cardiovascular Disease and Cancer Incidence. J. Pers. Med..

[44] Paulson J.C., Macauley M.S., Kawasaki N. (2012). Siglecs as Sensors of Self in Innate and Adaptive Immune Responses. Ann. N. Y Acad. Sci..

[45] Varki A., Angata T. (2006). Siglecs—The Major Subfamily of I-Type Lectins. Glycobiology.

[46] Journo G., Ahuja A., Dias-Polak D., Eran Y., Bergman R., Shamay M. (2021). Global CpG DNA Methylation Footprint in Kaposi’s Sarcoma. Front. Cell Infect. Microbiol..

[47] Anić P., Golubić Talić J., Božinović K., Dediol E., Mravak-Stipetić M., Grce M., Milutin Gašperov N. (2023). Methylation of Immune Gene Promoters in Oral and Oropharyngeal Cancer. Int. J. Mol. Sci..

[48] Li B., Zhang B., Wang X., Zeng Z., Huang Z., Zhang L., Wei F., Ren X., Yang L. (2020). Expression Signature, Prognosis Value, and Immune Characteristics of Siglec-15 Identified by Pan-Cancer Analysis. OncoImmunology.

[49] Ciccarese C., Massari F., Blanca A., Tortora G., Montironi R., Cheng L., Scarpelli M., Raspollini M.R., Vau N., Fonseca J. (2017). Tp53 and Its Potential Therapeutic Role as a Target in Bladder Cancer. Expert. Opin. Ther. Targets.

[50] Qiu Y., Ke S., Chen J., Qin Z., Zhang W., Yuan Y., Meng D., Zhao G., Wu K., Li B. (2022). FOXP3+ Regulatory T Cells and the Immune Escape in Solid Tumours. Front. Immunol..

[51] Shao L., Hou W., Scharping N.E., Vendetti F.P., Srivastava R., Roy C.N., Menk A.V., Wang Y., Chauvin J.-M., Karukonda P. (2019). IRF1 Inhibits Antitumor Immunity through the Upregulation of PD-L1 in the Tumor Cell. Cancer Immunol. Res..

[52] De Streel G., Lucas S. (2021). Targeting Immunosuppression by TGF-Β1 for Cancer Immunotherapy. Biochem. Pharmacol..

[53] Rathore V., Cheng C.-Y., Chen S.-P., Lin C.-Y., Chang C.-R., Lin W.-W. (2025). CASK Promotes Prostate Cancer Progression via Kinase-Dependent Activation of AKT. Int. J. Biol. Macromol..

[54] Szeto G.L., Finley S.D. (2019). Integrative Approaches to Cancer Immunotherapy. Trends Cancer.

[55] Wołącewicz M., Hrynkiewicz R., Grywalska E., Suchojad T., Leksowski T., Roliński J., Niedźwiedzka-Rystwej P. (2020). Immunotherapy in Bladder Cancer: Current Methods and Future Perspectives. Cancers.

[56] Xiang C., Chen J., Fu P. (2017). HGF/Met Signaling in Cancer Invasion: The Impact on Cytoskeleton Remodeling. Cancers.

[57] Breyer J., Wirtz R.M., Otto W., Erben P., Kriegmair M.C., Stoehr R., Eckstein M., Eidt S., Denzinger S., Burger M. (2017). In Stage pT1 Non-Muscle-Invasive Bladder Cancer (NMIBC), High KRT20 and Low KRT5 mRNA Expression Identify the Luminal Subtype and Predict Recurrence and Survival. Virchows Arch..

[58] Chen Y., Ji S., Ying J., Sun Y., Liu J., Yin G. (2022). KRT6A Expedites Bladder Cancer Progression, Regulated by miR-31-5p. Cell Cycle.

[59] Yu W., Yao D., Ma X., Hou J., Tian J. (2025). A Novel Efferocytosis-Related Gene Signature for Predicting Prognosis and Therapeutic Response in Bladder Cancer. Sci. Rep..

[60] Tai G., Ranjzad P., Marriage F., Rehman S., Denley H., Dixon J., Mitchell K., Day P.J.R., Woolf A.S. (2013). Cytokeratin 15 Marks Basal Epithelia in Developing Ureters and Is Upregulated in a Subset of Urothelial Cell Carcinomas. PLoS ONE.

[61] Plimack E.R., Hoffman-Censits J.H., Viterbo R., Trabulsi E.J., Ross E.A., Greenberg R.E., Chen D.Y.T., Lallas C.D., Wong Y.-N., Lin J. (2014). Accelerated Methotrexate, Vinblastine, Doxorubicin, and Cisplatin Is Safe, Effective, and Efficient Neoadjuvant Treatment for Muscle-Invasive Bladder Cancer: Results of a Multicenter Phase II Study With Molecular Correlates of Response and Toxicity. JCO.

[62] Appleman L.J., Beumer J.H., Jiang Y., Lin Y., Ding F., Puhalla S., Swartz L., Owonikoko T.K., Donald Harvey R., Stoller R. (2019). Phase 1 Study of Veliparib (ABT-888), a Poly (ADP-Ribose) Polymerase Inhibitor, with Carboplatin and Paclitaxel in Advanced Solid Malignancies. Cancer Chemother. Pharmacol..

[63] Lu L., Saha D., Martuza R.L., Rabkin S.D., Wakimoto H. (2015). Single Agent Efficacy of the VEGFR Kinase Inhibitor Axitinib in Preclinical Models of Glioblastoma. J. Neuro-Oncol..

[64] Matsuoka M., Komoike Y. (2015). Experimental Evidence Shows Salubrinal, an eIF2α Dephosphorylation Inhibitor, Reduces Xenotoxicant-Induced Cellular Damage. Int. J. Mol. Sci..

